# Improving the quality and efficiency of follow-up after curative treatment for breast cancer – rationale and study design of the MaCare trial

**DOI:** 10.1186/1471-2407-7-1

**Published:** 2007-01-02

**Authors:** Merel L Kimman, Adri C Voogd, Carmen D Dirksen, Paul Falger, Pierre Hupperets, Kristien Keymeulen, Marlene Hebly, Cary Dehing, Philippe Lambin, Liesbeth J Boersma

**Affiliations:** 1 Maastro Clinic, Maastricht, The Netherlands; 2 Department GROW – MAASTRO, Maastricht University, The Netherlands; 3 Department of Epidemiology, Maastricht University, The Netherlands; 4 Department of Clinical Epidemiology and Medical Technology Assessment, University Hospital Maastricht, The Netherlands; 5 Department of Medical Psychology, University Hospital Maastricht, The Netherlands; 6 Department of Medical Oncology, University Hospital Maastricht, The Netherlands; 7 Department of Surgery, University Hospital Maastricht, The Netherlands; 8 Department of Radiation Oncology, University Hospital Maastricht, The Netherlands

## Abstract

**Background:**

After curative treatment for breast cancer women frequently attend scheduled follow-up examinations. Usually the follow-up is most frequent in the first 2–3 years (2–4 times a year); thereafter the frequency is reduced to once a year in most countries. Its main aim is to detect local disease recurrence, or a second primary breast cancer, but also to provide information and psychosocial support. However, the cost-effectiveness of these frequent visits is under much debate, leading to a search for less intensive and more cost-effective follow-up strategies.

In this paper the design of the MaCare trial is described. This trial compares the cost-effectiveness of four follow-up strategies for curatively treated breast cancer patients. We investigate the costs and effects of nurse-led telephone follow-up and a short educational group programme.

**Methods/design:**

The MaCare trial is a multi centre randomised clinical trial in which 320 breast cancer patients are randomised into four follow-up strategies, focussed on the first 18 months after treatment: 1) standard follow-up; 2) nurse-led telephone follow-up; 3) arm 1 with the educational group programme; 4) arm 2 with the educational group programme. Data is collected at baseline and 3, 6, 12 and 18 months after treatment. The primary endpoint of the trial is cancer-specific quality of life as measured by the global health/QoL scale of the EORTC QLQ-C30. Secondary outcomes are perceived feelings of control, anxiety, patients' satisfaction with follow-up and costs. A cost-effectiveness analysis will be performed from a societal perspective.

**Discussion:**

Reduced follow-up strategies for breast cancer have not yet been widely applied in clinical practice. Improvement of psychosocial support and information to patients could lead to a better acceptance of reduced follow-up. The MaCare trial combines a reduced follow-up strategy with additional psychosocial support. Less frequent follow-up can reduce the burden on medical specialists and costs. The educational group programme can improve QoL of patients, but also less frequent follow-up can improve QoL by reducing the anxiety experienced for each follow-up visit. Results of the trial will provide knowledge on both costs and psychosocial aspects regarding follow-up and are expected in 2009.

## Background

With an estimated 1.15 million new cases worldwide each year, breast cancer is by far the most frequent cancer in women. Because of its high incidence and relatively good prognosis, breast cancer is also the most prevalent cancer in the world today [1]. After curative treatment for breast cancer women frequently attend scheduled follow-up examinations. Usually the follow-up is most frequent in the first 2–3 years (2–4 times a year); thereafter the frequency is reduced to once a year in most countries. The main objective of these examinations is to detect local disease recurrence or a second primary breast cancer in an early stage, hoping that this may increase the chances of cure. Follow-up should also provide information and psychological support. Another aim is to collect data on late effects of surgery, radiotherapy and chemotherapy for audit or research and to provide feedback to physicians [2,3]. However, there is much debate whether the objectives of breast cancer follow-up are adequately met in current practice [4,5]. Schapira (1993) reported that the early frequent outpatient clinic visits with only physical examination and an annual mammography do not improve early detection of recurrence or overall survival [6]. Since then, several studies have investigated the effect of the frequency [7] and intensity [8,9] of follow-up on overall survival, without finding evidence supporting the necessity of frequent or intensive follow-up. Furthermore, instead of providing psychosocial support, it has been demonstrated that the outpatient clinic visit may induce anxiety because of the risk of detecting tumour relapse [10,11].

Although the current frequent follow-up strategies thus seem to miss their most important goals, they do depend heavily on expensive and scarce specialised knowledge for routine history taking and physical examination. Financial constraints have forced oncologists and policy makers to search for alternative and cost-effective follow-up strategies. Studies on follow-up by the general practitioner, patient-initiated or nurse-led follow-up or contact by telephone showed no difference in time to detection of recurrence or patients perceived quality of life (QoL) [12-16]. Nevertheless, these reduced strategies have not yet been widely accepted and applied in clinical practice. This may on the one hand be ascribed to the perception of many medical specialists that most patients need reassurance and psychosocial support, and on the other hand to the false expectations of many patients that frequent follow-up will result in a better overall survival [17]. Improvement of the psychosocial support and education of the patients may lead to a better acceptance of both patients and medical specialists of a reduced follow-up policy. A number of studies have been performed using various group intervention formats for breast cancer patients to provide psychosocial support. However, most interventions mainly focussed on intensive psychological support (10 sessions or more), showing varying results and often high refusal rates [18].

No trials have been performed that combine a reduced follow-up policy in the first 2 years with an acceptable strategy to provide information and education. For this reason, two interventions for early breast cancer follow-up were developed for the study that is discussed in this paper; nurse-led telephone follow-up and a short educational group programme (EGP). The primary aim of the so-called MaCare trial (Mamma carcinoma and afterCare) is to investigate the impact of these interventions on cancer-specific QoL and costs and thus to determine which of these follow-up strategies is the most cost-effective. This paper reports on the methodological design of the trial as well as the contents of the interventions and the economic evaluation.

## Methods/Design

### Study design

**Figure 1:**
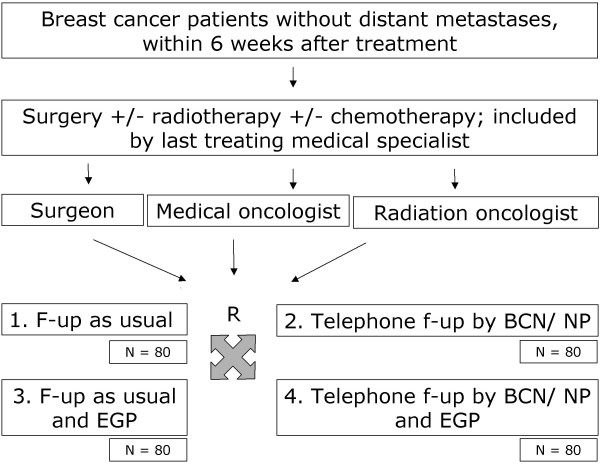
Study design of the MaCare trial 'Improving the quality and efficiency of follow-up after curative treatment for breast cancer'. F-up = follow-up; R = randomisation; EGP = educational group programme; BCN = breast care nurse; NP = nurse practitioner; N = number of patients

The MaCare trial is a multi centre randomised trial, in which breast cancer patients who are treated with curative intent are randomised between four follow-up strategies (see figure 1):

1) Follow-up as usual; 5 outpatient clinic visits in the first 18 months (at 3, 6, 9, 12, and 18 months), with a mammography at one year.

2) Nurse-led telephone follow-up; a mammography at one year combined with an outpatient clinic visit, and telephone interviews by a breast care nurse (BCN) or nurse practitioner (NP) at the same time points as during the usual follow-up (i.e. 3, 6, 9 and 18 months).

3) Similar to arm 1, combined with the EGP.

4) Similar to arm 2, combined with the EGP.

After 18 months patients return to the current follow-up strategy, i.e. follow-up once a year.

### Setting

The trial is carried out in seven hospitals and two radiotherapy clinics located in the south of The Netherlands. Medical specialists working in the participating centres have been recruiting patients since July 2005 and will continue recruitment until approximately September 2007. Follow-up as usual takes place in the hospital where surgery and chemotherapy were performed, alternating between the surgeon, medical oncologist and radiation oncologist. The telephone follow-up is performed by the nurse practitioner (NP) or breast care nurse (BCN) working at this hospital. An NP is a registered nurse who has acquired (at masters level) the expert knowledge base, complex decision-making skills and clinical competencies for expanded practice. The BCN is a qualified nurse who has had specialist training in breast care and who guides the patient throughout treatment. In this paper the term BCN is used and refers to both types of nurses. The EGP is held at cancer information centres. These centres are specialised in providing support to (ex-)cancer patients and their relatives through education and organising activities. Selecting participants from multiple centres and locations in the Netherlands allows us to include a representative selection of breast cancer patients. Participation is voluntary and after written informed consent is obtained. Prior to the start of the study the protocol was approved by the Medical Ethical Committee of MAASTRO Clinic. Furthermore, all participating centres signed a declaration on local feasibility before inclusion of their first patient, according to the Dutch law and regulations.

### Study population

#### Selection of participants

Patients are recruited and included within 6 weeks after completion of treatment with curative intent by the medical specialist of the last treatment modality. Eligibility criteria are summed up in table 1. If eligible, the medical specialist introduces the MaCare trial to the patient and gives her a detailed information sheet. Within two weeks, the research assistant contacts the patient to explain the aim and implications of the trial again. Eligibility criteria are checked once more and the research assistant asks whether the patient wants to participate.

#### Non-participants

Patients who are eligible for inclusion but not interested in participation are asked to fill out the same baseline questionnaires (see outcome parameters) as participants. By assessing whether there are differences between participants and non-participants the external validity of the study results can be determined.

#### Sample size calculations

The primary outcome measure is cancer specific QoL at 12 months after randomisation, measured by the global health/QoL scale of the EORTC QLQ-C30. A baseline score of 64 with a standard deviation of 17 [19,20] is considered as a starting point. In an analysis of the clinical significance of changes in QoL, Osoba et al (1998) showed that patients judge a change between 5–10 to be small, between 10–20 to be moderate, and more than 20 to be large [21]. Consequently, we considered a change smaller than 5 points to be no change. To show that QoL of patients in arms 2 and 4 (telephone follow-up) is at least similar to patients in arm 1 and 3 (usual follow-up) with a power of 80% and a significance level of 0.05, the required number of patients per group is 80. With these 320 patients an improvement in QoL, by adding the EGP compared to no EGP, of 10 points with a power of 0.95 and a significance level smaller than 0.01 can be detected. A dropout rate of 10% is accounted for in these calculations.

### Randomisation

After written informed consent and completion of the baseline questionnaire, randomisation by minimisation is performed by the Comprehensive Cancer Centre Limburg. Patients are pre-stratified by treatment modalities and hospital. After randomisation, the research assistant contacts the patient and informs her about the assigned study-arm, thus whether she will receive standard or telephone follow-up and whether she will be invited to join the EGP.

### Interventions

#### Usual care

The first study arm, the standard arm, consists of follow-up as described in the Dutch guidelines. After treatment, the patient has an outpatient clinic visit with the medical specialist or BCN every 3 months during the first year, every 6 months during the second year, and thereafter once a year until at least 5 years. Mammography is taken once a year.

#### Nurse-led telephone follow-up

In study arms 2 and 4 a mammography is taken once a year, which is combined with a visit to the medical oncologist, the surgeon, BCN or the radiation oncologist. At the other regular follow-up times (i.e. at 3, 6, 9, and 18 months), the BCN has a telephone interview with the patient. The telephone interview is done by open discussion and a semi structured questionnaire, including screening for physical – especially loco regional – and psychosocial symptoms, and compliance to hormonal therapy. Data on the side effects of treatment are also collected during the interview. Furthermore, the BCN informs about general well being of the patient, her family-life, relationships and work reintegration. When the patient does not feel reassured an additional appointment is made for her to come to the hospital. The telephone follow-up is mainly meant to reduce the workload of medical specialists, with possible reduction of costs, but could also influence QoL as a result of a reduction of stressful visits to the outpatient clinic [11]. To adequately perform the telephone interview, all participating BCNs attained a training, specifically developed for this study. In this training nurses are informed on the most recent developments in breast cancer treatment and follow-up, develop a semi-structured questionnaire for support during the interview, and practice their telephone communication skills with a breast cancer patient.

#### Educational Group Programme

In arms 3 and 4 of the study, the patient and their partner (or close friend or family member) are invited to participate in the Educational Group Programme (EGP) within three months after treatment. The basic theory underlying the EGP is the transaction process theory of stress [22]. The group-format programme focuses on the first two stages of the transaction process theory of stress, the primary and secondary appraisal. The EGP therefore consists of two interactive group sessions of 2,5 hours of patient education. In the first session, which addresses the primary appraisal, a BCN provides information on possible side effects of the different treatments and issues such as prostheses, exhaustion, hereditary issues and financial consequences. Also, signs and symptoms of recurrences are discussed. A health care psychologist concentrates on psychological consequences, like anxiety, depression and changed family role patterns. The second session focuses on the second appraisal, i.e. information is provided on possible coping strategies and actions that could be undertaken when symptoms occur. Furthermore, information on the various health care workers and additional interventions is given. Discussions are stimulated by the speakers, addressing issues such as how to deal with anxiety, mood changes and changes in relationships. In this way, patients and their partners are stimulated to be alert to symptoms, and to enhance self-efficacy in adopting one of the proposed actions or coping strategies. It is expected that this will lead to a better QoL. During the second session a volunteer from the Dutch Breast Cancer Association presents regional activities available for participants. The EGP is completed with a relaxation exercise. After the two sessions, participants receive a small booklet encompassing the topics discussed in the programme. The BCN and health care psychologist use standardised presentations, so that the EGPs given during the time of this study are as similar as possible. Furthermore, by using standard presentations, different teams can lead the EGP and the programme will be easily transferable to other settings.

### Primary and secondary outcome measures

#### Primary outcome measure

The primary domain for improvement for both interventions in the trial is cancer-specific QoL. Cancer-specific QoL is measured by the QoL scale of the European Organisation for Research and Treatment of Cancer (EORTC) QLQ-C30. The EORTC QLQ-C30 has a validated Dutch-language version available [23].

#### Secondary outcome measures

Secondary outcome measures are perceived behavioural control, anxiety, patients' satisfaction with follow-up, generic health-related quality of life and societal costs. Perceived behavioural control is measured by the Dutch version of the Mastery Scale [24]. A question whether the patient is experiencing emotional support from partner (if applicable) is added. Anxiety is measured by the validated Dutch version of the State-Trait Anxiety Inventory (STAI) [25]. Patients' satisfaction with follow-up will be assessed using the validated Patient Satisfaction Questionnaire – NL (PSQ-NL) [26]. Generic health-related quality of life is measured by the EuroQol-5D (EQ-5D) [27].

Societal costs are determined by the hospital information systems and prospective cost-diaries. More details on the costs are provided in the economic evaluation section.

#### Time schedule

Patients complete questionnaires at baseline, and at 3, 6, 12 and 18 months intervals. Costs-diaries are filled out by the patients over a four week period, at the same time points. The questionnaires and cost-diaries are sent by mail. If not returned within 14 days, patients are contacted by the research assistant and are asked to continue participation.

### Statistical analyses

To evaluate differences in QoL outcome between the study arms, the data will be analysed using a multivariate linear regression model. In this model both the EGP factor and the telephone factor will be taken into account. In addition, variables such as age, hospital treatment, and experienced support of a partner, will be brought into the model. The procedure will be performed 4 times as QoL is assessed at 3, 6, 12 and 18 months. Differences in satisfaction with care, perceived behavioural control and anxiety will be analysed in the same way. Participation at group meetings will be analysed using logistic regression models. Differences in medical consumption will be evaluated using a multivariate linear regression model. Subgroup analysis will be performed to identify specific patient characteristics that could predict the optimal follow-up strategy for the individual patient. Data will be analysed according to the intention-to-treat principle.

The number of recurrences is expected to be very low with a follow-up of 18 months [28]. Statistical analysis of the difference in time-to-detection per group will therefore be limited to descriptive statistics.

### Economic evaluation

Parallel to the trial an economic evaluation will be performed, comparing the costs and effects of each separate follow-up strategy, in order to determine the most cost-effective follow-up. The cost-effectiveness comparison thus includes four different strategies corresponding to the four study-arms. The cost-effectiveness ratio(s) will be expressed as the incremental cost per quality-adjusted life year (QALY). In order to be able to construct a QALY, the scores on the 5 dimensions of the EuroQol [29] are used. The cost-effectiveness analysis is performed from a societal perspective. Sensitivity analyses will subsequently be performed on several variables. For future costs and effectiveness data, a discount rate of 4% will be handled. In order to quantify the uncertainty surrounding the costs and effectiveness results, bootstrap analyses will be performed.

The cost-analysis from a societal perspective includes all health care costs and costs outside the health care sector. The cost-analysis will be performed by means of the micro-costing method, whereby a detailed inventory of the resource use per patient will take place [30]. Costs are calculated by multiplying resource utilisation with the cost-price per unit. Health care costs refer to hospital costs and costs of other health care facilities. Hospital costs consist of personnel, material, capacity costs and overhead associated with the four programmes and costs of (scheduled and unscheduled) outpatient hospital visits, telephone interviews and medical services (e.g. laboratory tests, bone scans, etc.). Most of these costs are estimated by using existing resource registration systems and available cost prices through the financial departments of the participating centres. In case true cost prices are not readily available in all participating centres the existing cost-prices of the University Hospital Maastricht or official directive prices are used [31]. Costs of telephone follow-up consist of personnel costs of the BCN and telephone costs. Costs associated with the EGP primarily consist of the personnel costs. Other health care costs include costs of visits to the general practitioner, medication, psychological and/or psychiatric (emergency) care.

Costs outside the health care sector refer to out-of-pocket expenditures and productivity costs due to reductions in paid work and/or domestic activities. These costs are determined by a prospective cost diary, which is filled in by the patient at baseline, and thereafter at 4 time points (3, 6, 12, and 18 months after treatment) for a period of 4 weeks. Productivity losses due to absence from work are estimated by means of the friction cost method [31,32]. In case of domestic or other unpaid activities, shadow prices will be used.

## Discussion

Current frequent follow-up is not meeting its intended aims, but does raise the burden on medical specialists, leading to high medical costs. Although studies investigating reduced follow-up have shown that there is no difference in time to detection of recurrence and patients perceived QoL, reduced follow-up has not yet been widely accepted and applied. Medical specialist may feel that patients need constant reassurance and patients have the false expectation that frequent follow-up will improve survival [17]. Improvement of psychosocial support and information to patients could lead to a better acceptance of reduced follow-up. The trial reported in this paper combines these two aspects. By performing an economic evaluation parallel to the trial, the ultimate aim is to find an alternative and more cost-effective follow-up strategy. We expect the educational group programme to help breast cancer patients and their partners in dealing effectively and efficiently with the consequences of a recent diagnosis and treatment of a potentially fatal disease. This increase in perceived behavioural control is expected to improve QoL. The nurse-led telephone calls are expected to render visits to the outpatient clinic superfluous, which may subsequently reduce the anxiety experienced by patients for each follow-up visit. Looking at costs, the telephone follow-up is expected to be cost-saving, due to fewer visits to the medical specialist. The EGP is expected to result in a slight cost-increase per patient in the first follow-up year, as compared to standard follow-up. However, due to the expected increase in QoL it may yield 1) reduced health care costs and 2) possibly lower costs due to a reduction in productivity losses.

The close cooperation with many centres enables us to reach a representative study population of women treated for primary breast cancer and enhances the generalisability of the trial results. In addition, BCNs are systematically trained to perform telephone follow-up and the EGP is based on standardised presentations. If one or both interventions appear to be successful, they are easily transferable to other settings. Finally, results may be extended to other cancer patients, but a further study would be required.

## Competing interests

The author(s) declare that they have no competing interests.

## Authors' contributions

MLK is responsible for data collection and drafted the manuscript. MLK works under direct supervision of CDD and LJB. ACV, PF, MH, CDD, PD, CD, LJB en PhL all participated in discussing the design of the study and developing the research protocol. They were co-applicants on the successful funding proposal. All authors read and corrected draft versions of the manuscript.

## Pre-publication history

The pre-publication history for this paper can be accessed here:



## Figures and Tables

**Table 1:** Study inclusion and exclusion criteria

*Inclusion criteria* • Female patients with breast cancer without distant metastases • WHO performance scale 0–2 • Treated with curative intent (i.e. lumpectomy or mastectomy, with or without radiotherapy and/or chemotherapy) • Treatment completed < 6 weeks prior to randomisation • Able to speak and read fluently in Dutch	
*Exclusion criteria* • Participation in another trial, requiring more frequent follow-up • Medical diseases (treatment-related side-effects, concomitant tumours) requiring frequent follow-up	
